# Anatomic Study of the Retaining Ligaments of the Face and Applications for Facial Rejuvenation

**DOI:** 10.1007/s00266-013-0066-8

**Published:** 2013-03-14

**Authors:** Bryan C. Mendelson

**Affiliations:** The Centre for Facial Plastic Surgery, 109 Mathoura Rd, Toorak, VIC Australia

*Level of Evidence V* This journal requires that authors assign a level of evidence to each article. For a full description of these Evidence-Based Medicine ratings, please refer to the Table of Contents or the online Instructions to Authors http://www.springer.com/00266.

Any article entitled “Anatomic Study of the Retaining Ligaments of the Face” [1] is certain to attract the attention of plastic surgeons who operate on the face. Surgeons want to know about the facial structure, even though 20 years has elapsed since the original description of the ligaments that explain the fixation of the facial soft tissues to key bony landmarks [2, 3].

Opinion remains divided on how to best use the superficial musculoaponeurotic system (SMAS) and indeed how to best use the ligaments. Surgeons, including Furnas [4], the current authors, and others, operate on the surface of the SMAS, while other surgeons operate in the “deep plane,” the glide plane under the SMAS [5]. Either way, the SMAS is used to directly support (lift, tone, and reshape) the overlying tissues, while the ligaments support the SMAS.

Being a member of the “superficial-to-the-SMAS school,” the authors give as the reason for this anatomical study “differences” between the few studies related to the location of the ligaments, although they do not stipulate what these differences are. This may explain the limited focus of their cadaver study, which was mainly to determine the location of the two major ligaments, the zygomatic and masseteric, by measurement from the tragus, the reference point clinically, being from the incision. In reality, both the zygomatic and masseteric ligaments are not solitary structures but a series of ligaments, and for the purposes of their study, the most anterior part of where the ligament reinforces the SMAS is the point they recorded. Although they defined the location of the ligaments, they did not discuss the significance of their dissection results.

Accordingly, despite the name of the study, it does not add new information to the literature on facial ligaments. However, it does provide some excellent photographs of the dissection that are beneficial for review. The photographs of the deeper, sub-SMAS dissections, Figs. 6, 7, and 8, should be most useful for readers and warrant study.

The prezygomatic space is shown very clearly in Fig. 6, as it underlies the orbicularis oculi roof of the space. The upper and lower ligamentous boundaries, the orbicularis retaining ligament, and the zygomatic ligaments are seen and, for those who have not been convinced about the existence of this space, the interval between the ligamentous boundaries is shown [6]. This interval was shown by this anatomical study to be 2 cm on average. Utilization of the prezygomatic space is the focus of Aston’s FAME (finger-assisted midcheek elevation) technique, and the dissection seen in the study’s Fig. 6 shows the space where the finger is placed and where it dissects forward in the FAME technique [7]. The space is then seen from a different perspective, i.e., from the medial aspect in Figs. 7 and 8. All surgeons, including those who do not perform the FAME procedure, need to appreciate this anatomy because laxity of the roof of this space is the basis for malar mounds and for their correction.

In the clinical part of their work, applying their understanding of the anatomy, Rossell-Perry and Paredes-Leandro perform their facelift dissection in two different planes depending on the part of the face. Over the infrazygomatic part of the face, the dissection plane is deep subcutaneous on the surface of the SMAS. Whereas, overlying the zygoma above, the dissection is deep to the SMAS. (This is the FAME dissection in the prezygomatic space deep to the orbicularis.) The authors’ description of this is confusing. The dissection in the space is deep to the SMAS and, on continuing the dissection medial to the space the level of the dissection remains beneath the SMAS where the malar fat pad overlies the SMAS, not in the subcutaneous plane, as described.

Of course, the results of a facelift are determined by the suture fixation, not the dissection itself. The purpose of the selective release of ligamentous fixation is to maximize the mobility obtained by the dissection [8]. The authors tighten the laxity of the SMAS anterior to each of the three ligaments by suturing back to the fixed SMAS (i.e., where the SMAS is stabilized by its skeletal fixation via each of the three mentioned ligaments). For some reason they used an exceptionally delicate suture for this fixation, which is inconsistent with the inherent strength of the ligament to which it is being sutured. The natural strength of the ligaments gives an indication of the forces they have to resist. In effect, only three delicate fixation sutures were used in the infrazygomatic region in an endeavor to support the face. This is expecting a lot.

In contrast, with the sub-SMAS dissection in the prezygomatic region (the upper cheek), there is not any mention of fixation of the SMAS at all, even though the prezygomatic space has been dissected. The absence of tightening and fixation here could well explain the lack of correction of the lid cheek area in their results.

**Fig. 1:**
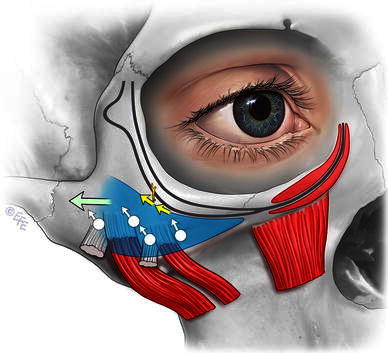
Location of the fixation sutures in the prezygomatic space and their vectors

**Fig. 2:**
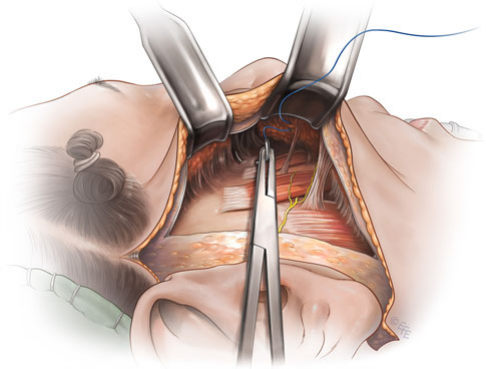
Technique of SMAS fixation suture placement in the prezygomatic space

Since learning of the anatomy of the prezygomatic space over 10 years ago, in my practice I enter the space to place a series of 3/0 permanent braided sutures (ligament-like) through the boundaries of the prezygomatic space (Figs. 1, 2). The first group of sutures is along the lower boundary to support the SMAS of the upper nasolabial fold and mimic the effect of the medial zygomatic ligaments. The second group of sutures is to tighten the SMAS in the upper boundary (formed by the orbicularis retaining ligament), with some ligament release as necessary. This provides tone to the lower lid and lid–cheek junction. Finally, a transversely oriented suture is placed near the lateral border of the prezygomatic space to retone its roof.

The complications reported in the series were related mainly to the wide skin undermining; they were hematoma (20 %, 4 % major and 16 % minor) and prolonged loss of sensation (30 %). You would not expect to have permanent facial nerve palsy with this procedure, but the 7 % incidence of temporary palsy is greater than would be expected for a superficial dissection. There is no information given about which branches of the facial nerve were affected to know whether these palsies resulted from the prezygomatic space dissection (zygomatic branch) or from the pre-SMAS dissection which could involve the buccal branches. Hopefully, the temporal and mandibular branches were not involved in their dissection.

Despite these issues, the results of the authors’ patient satisfaction survey were very positive, with a 100 % “yes” response to the question of whether the result was maintained at 1 year, and over 90 % considered the result to be natural and would recommend the procedure to others.

The regions of the face where the results were best, according to the patients, were the midface and jawline, with an impressive 30+ % with their expectations exceeded. The least satisfaction was with the correction of the nasolabial fold area, with 17 % responding that the results were less than “very good” (minimal or modest improvement). This is the usual experience with facelifts and these figures give the authors direction in which to improve their facelifts.

I appreciate the authors’ comprehensive approach to understanding the facelift. Although this is not a breakthrough contribution, the evolution of their technique and its assessment are clearly described. If I may make some suggestions: To reduce complications and improve results, first reduce the extent of skin undermining. Minimizing skin undermining is an inherent advantage of the Composite facelift and there is minimal bleeding and bruising as well as a more robust flap [9]. The second suggestion is to focus on improving the correction of the upper nasolabial fold. This could be done by a proper SMAS tightening perpendicular to the fold, as shown in Figs. 1 and 2, along with volume replacement of the skeletal resorption of the maxilla underlying the nasolabial groove, to improve their already high levels of patient satisfaction.
